# Bilateral Double-headed Recurrent Pterygium: A Case Presentation and Literature Review

**Published:** 2020-03-15

**Authors:** Bengi Ece Kurtul, Ahmet Kakac, Abdulkerim Karaaslan

**Affiliations:** 1 Department of Ophthalmology, Mustafa Kemal University Tayfur Ata Sokmen Faculty of Medicine, Hatay, Turkey

**Keywords:** Pterygium, Double-headed, Recurrent, Bilateral, Conjunctival Autograft Transplantation.

## Abstract

Pterygium is a frequent corneal disease characterized by growing of fibrovascular tissue from the bulbar conjunctiva onto the cornea. Although the causes of pterygium are not obvious, sun exposure is closely correlated with its development. Pterygium, especially double-headed pterygium is mostly seen in warm climate and in individuals who work outdoors. A minority of pterygium is double-headed (both temporal and nasal origins). Bilateral recurrent double-headed pterygium is a very rare condition. Here, we reported a 35-year-old male patient with bilateral recurrent double-headed pterygium. A brief review about the recent literature concerning the etiology, associated risk factors, operation types and management of patients with recurrent pterygium was also discussed. The patient was a field worker, smoker and had a family history of pterygium. There was no ocular surgery history except pterygium surgery 15 years ago in both eyes. A successful pterygium excision was performed under local anesthesia with the vertical split conjunctival autograft transplantation (CAT) in both temporal and nasal parts of the right eye (randomly) for cosmetic disfigurement and avoidance of recurrence. The pathological report was consistent with pterygium. At postoperative first week, first and 1.5^th^ month visits, conjunctival autografts were in place and stable. No complications such as infection, corneal thinning or graft dislocation were seen. The patient was satisfied with his right eye and demanded the same surgery for his left eye. We suggest vertical split simultaneous CAT as a safe and useful surgical method for the treatment of recurrent double-headed pterygium; however, longer follow-up is required to confirm the outcome.

## INTRODUCTION

Pterygium is one of the leading causes of blindness and visual disorder [1, 2]. It is known as a widespread fibrovascular wedge-like lesion of the epithelial tissue invading cornea [3-8]. Pterygium usually occurs due to chronic ultraviolet exposure and develops at the nasal part of the cornea (97%) [3]. A rare type of pterygium is double-headed (both temporal and nasal origins) and bilateral form which is scarcely seen [3]. There is only one paper in the literature regarding pterygium-like corneal alterations of three patients in one family complained from persistent recurrences with bilateral symmetrical involvement of the nasal and temporal cornea [9]. Double-headed pterygium contradicts the surgeon regarding simultaneous or two-stage surgery [3, 4]. Different surgical techniques for treatment of double-headed pterygium have been applied [3, 4]. Perfect pterygium surgery should achieve a low or no recurrence rate, negligible complications and good cosmetic outcome [3, 4]. 

In this manuscript, an interesting rare case of a bilateral double-headed recurrent pterygium and vertical split conjunctival autograft transplantation (CAT) simultaneous surgery (to both nasal and temporal parts of the right eye) was presented. A brief review about the recent literature published in Pubmed without language and time restrictions concerning the etiology, associated risk factors, operation types and management of patients with recurrent pterygium was also discussed.

## CASE REPORT

**Figure 1:**
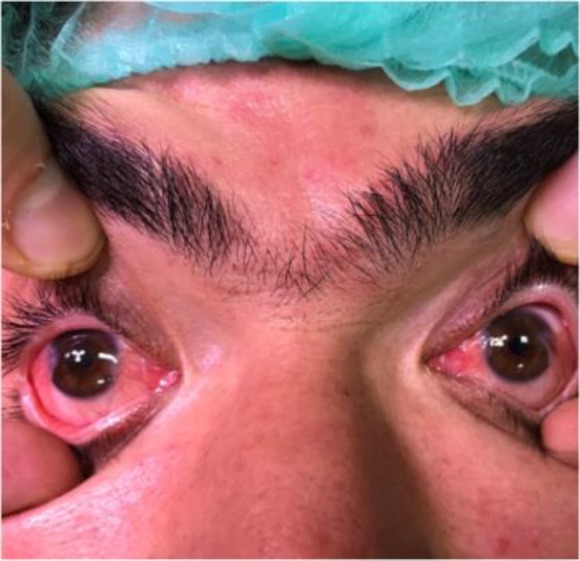
Double-Headed Bilateral Recurrent Pterygium at His First Presentation to Our Clinic

**Figure 2:**
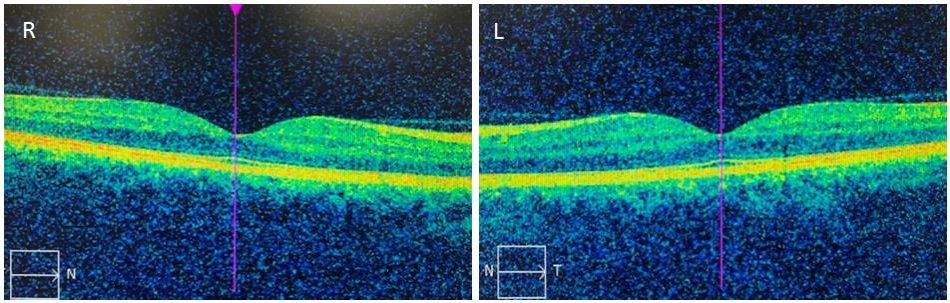
Optical Coherence Tomography Images of Both Eyes With Normal Appearance of Both Macular Area

A 35-year-old male patient, referred to our clinic for his bilateral recurrent double-headed pterygium for 15 years. He was a field worker, smoker and had a family history of pterygium. He told that his mother and sister had also pterygium. His visual acuity was full in both eyes. On slit-lamp examination, he had bilateral recurrent double headed pterygium. Slit photo of the patient was taken (Figure 1). In the right eye, recurrent pterygium in temporal side was larger than the nasal side, unexpectedly. The other ophthalmic findings were unremarkable. Schirmer test was also applied and both eye values were 30 mm. Intraocular pressures were 16 mmHg by noncontact tonometer in both eyes. Posterior segment exams had normal findings. Optical coherence tomography images of both eyes were shown in Figure 2. He had no history of any ocular surgery except for pterygium surgery 15 years ago in both eyes. He did not remember the method of previous pterygium surgery and his past medical documents were not available. He had no history of ocular trauma, systemic disease or medication. Routine laboratory findings revealed positive results for hepatitis B surface antigen. We referred the patient to the gastroenterology department for further evaluation and management of his systemic diagnosis. 

**Figure 3:**
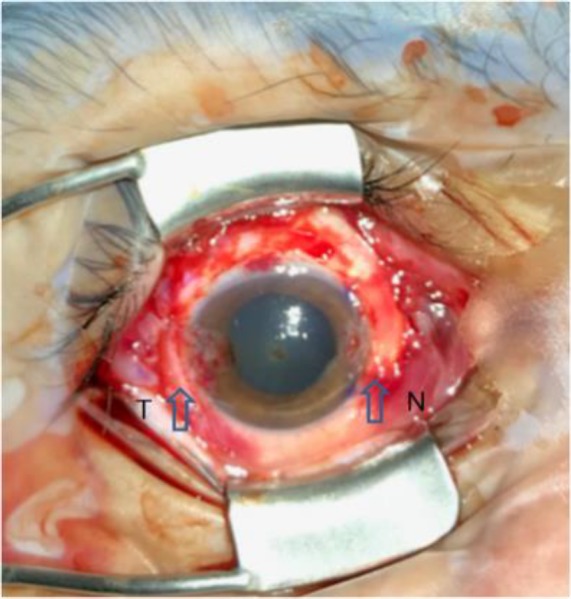
Patient’s Right Eye at the End of Operation. (N: Nasal; T: Temporal. Arrows show conjunctival autografts in place

**Figure 4:**
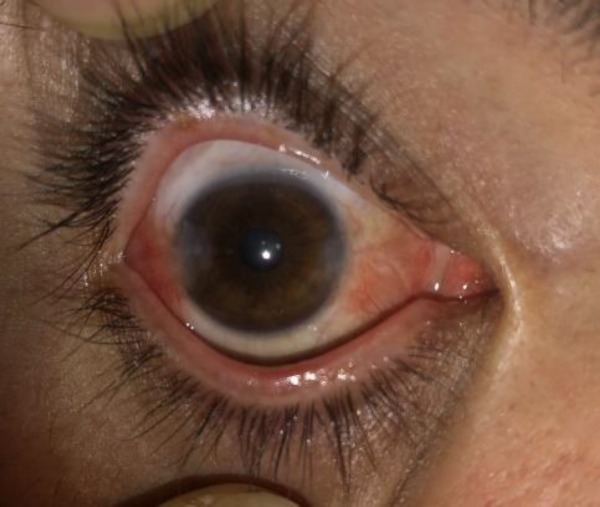
Slit-lamp photograph of the patient’s right eye at the postoperative 1.5-month, showing fine corneal opacity in nasal and temporal sides of peripheral cornea. But, no complications such as infection, corneal thinning or graft dislocation were seen

We decided to apply surgery randomly to the right eye a week later following consulting the patient. Successful excision for double-headed pterygium was made under local anesthesia in the right eye after receiving a signed informed consent. Following instillation of topical proparacaine hydrochloride 0.5% (Alcaine; Alcon Canada, Couvreur, Belgium), the sterile preparation and draping were applied to the right eye, and then a lid speculum was inserted. The bodies of the nasal and temporal pterygium were marked. Temporal pterygium was larger and excised firstly. Subconjunctival lidocaine hydrochloride + 2% epinephrine was injected into the pterygium head. The pterygium was excised from the cornea by using toothed forceps and crescent blade. Complete dissection of the pterygium body from the sclera, subconjunctival Tenon’s tissue and surrounding fibrovascular tissues was performed with the Westcott scissors. Homeostasis was achieved without using cauterizing. The conjunctival edge was trimmed. The corneal surface was gently scraped with a scalpel. After performing the same procedure on the nasal pterygium, a single piece of conjunctival tissue was taken from the superior bulbar conjunctiva of the same eye and divided into two pieces vertically based on the area of the removed pterygium and placed leaving no open scleral tissue starting from the nasal and temporal limbus, with the epithelial side up and the limbal area aligned with the limbus. Drugs to avoid recurrence like mitomycin C or 5-fluorouracil were not used. Both grafts were sutured to the surrounding conjunctiva by interrupted 7/0 vicryl. The excised pterygium specimen was sent for pathological investigation. Photograph of the right eye was taken at the end of operation (Figure 3). Following operation, therapeutic bandage contact lens was placed. Postoperatively, the patient received 0.5% loteprednol etabonate + tobramycin (Zylet, Bausch and Lomb, Rochester, NY, The USA) 4 times a day, bacitracin + neomycin sulfate ophthalmic ointment (Thiocilline, Abdi İbrahim, İstanbul, Turkey) twice daily, lubricant eye drop (Systane, Alcon Laboratories, Inc., Fort Worth, TX, The USA) for 10 days. In addition, 0.05% cyclosporin A (Restasis, Allergan Pharmaceutical, Irvine, CA, The USA) twice daily was prescribed for 6 weeks postoperatively. Patient was recommended to avoid dusty and sunny environments and to use sunglasses. The pathological report was consistent with pterygium. At the postoperative first week, first and 1.5-month visits, conjunctival autografts were in place and stable. No complications such as infection, corneal thinning or graft dislocation were seen during the 1.5-month follow-up. Therapeutic bandage contact lens was removed at the end of first month. He was satisfied with his right eye and demanded the same surgery for his left eye. Postoperative 1.5-month clinical photograph was shown in Figure 4.

## DISCUSSION

This patient was presented because of the rare incidence of bilateral, recurrent and double side corneal involvement. A successful pterygium excision was performed under local anesthesia with the vertical split CAT to both temporal and nasal parts of the right eye for cosmetic purposes and avoiding recurrence. 

Ethnicity is associated with development of pterygium. Chen T et al. [10] showed that Han ethnicity is a significant risk factor of pterygium. In other studies, the primary risk factors for pterygium were reported as age, male sex, ultraviolet exposure, outdoor work and smoking [11-23]. Pterygium has been reported also as a work-related disorder [24]. Our patient was a male field worker and smoker living in Hatay (a sunny city located in the southernmost of Turkey). In contrast to above studies, unexpectedly, outdoor occupations were not found as a risk factor for pterygium in the study of Akinsola FB et al. [25]. Malekifar P et al. [26] demonstrated that family history of pterygium and blepharitis found to be risk factors. Our patient had also a positive family history, but no blepharitis. Roka N et al. [27] mentioned a strong association between dry eye and pterygium. Dry eye was not an accompanying entity in our patient.

Double-headed pterygium enforces the surgeon about a simultaneous or two-stage surgery [3, 4]. Different surgical techniques for double-headed pterygium have been performed [3, 4]. Perfect pterygium surgery should achieve a low or no recurrence rate, negligible complications and good cosmetic purpose [3, 4]. CAT technique with a recurrence rate of 5% has been the most preferred one used by ophthalmologists for years [3-5], which was applied for our patient. In another study authors mentioned that sutured limbal conjunctival autografts had a recurrence rate ranging from 0% to 14.29% [9]. Dividing the free conjunctival autograft into two parts and suturing in place of the excised pterygium on both side of the corneas seems to be a good choice in double headed pterygium [3].

Yeung SN et al. [5] performed sequential pterygium excision with CAT in the management of double-headed pterygium. They mentioned that CAT from the same site several months later does not appear to increase the rate of recurrence and sequential CAT is a safe and effective method of addressing double-headed pterygium [5]. Our patient was a young man and had a cosmetic worry; additionally, he had bilateral recurrent double-headed pterygium. It would take time to recovery and he did not want to waste time for each surgery for both eyes.

Aidenloo NS et al. [6] suggested that young age, recurrent type of pterygium, and larger pterygial tissue are risk factors for pterygium recurrence after surgical excision. The recurrent pterygium was larger in temporal side in our patient’s right eye unexpectedly. They also recommended early excision of pterygium to reduce the recurrence rate. In the study of Lee JS et al. [7] authors reported that pterygium excision with a large conjunctival autograft for the treatment of recurrent pterygium provided an excellent cosmetic result, a low recurrence rate and minimal complications.

Anti-fibroproliferative application with 5-fluorouracil or mitomycin C may be a useful adjunct in primary pterygium surgery [28, 29]. However, we did not prefer anti-fibroproliferative application because of double sided pterygium. In a study, no significant recurrence rate was found between the application of sutures and fibrin glue for CAT [30]. We had no fibrin glue in our clinic available. Therefore, we used 7/0 vicryl sutures for CAT. Multi-layer amniotic membrane graft for pterygium was also mentioned [31]. Pterygium in our patient was not in an advanced size; therefore, we did not need to use multi-layer amniotic membrane.

Corneal perforation related with dellen is a rare but important complication of a pterygium excision. A 60-year-old man with these complications was reported by a bare sclera technique without adjunctive therapy [32].  CAT is reliable, uncomplicated, fast surgery without loss of tissue and prevents recurrence of pterygium. It also decreases the risk of granuloma formation, scleral thinning and necrosis [33, 34]. Deep lamellar keratoplasty with conjunctival autograft for recurrent advanced pterygium correlated with corneal thinning was mentioned [35]. Corneal thinning or any other complication was not seen by CAT in our patient 1.5-month postoperatively.

Pterygium formation was found strongly associated with its location and laterality [36]. our patient had a bilateral pterygium and did not have amblyopia or eccentric fixation. A case of a free cyst of iris, bilateral pterygium and symmetrical nodosity of the auricle has been previously published [37]. There were no other ophthalmic or systemic pathologies in our case.

Greater protrusion degree might be a risk factor for occurrence of pterygium [38]. Here, the greater protrusion was not seen and there was no advanced form of pterygium in our patient. Postoperative medication is important and instillation of 0.05% cyclosporine is safe and efficient [39]. We also offered patient to use postoperative 0.05% cyclosporine for 6 weeks. Wearing of face caps/hats and protective goggles were found to have a protective effect [40, 41]. Patient was recommended to abstain from sunny conditions and to use sunglasses.

To the best of our knowledge, this was the second case presentation of bilateral double double-headed pterygium with successful short term management. We had some limitations. Firstly, the pterygium excision for the left eye has not been planned yet and post-operative follow-up period is not long enough. It would be great if we could present both eyes after surgery and long follow-up. Secondly, a bilateral pterygium was presented in a 50-year-old male with H syndrome [42], patients with xeroderma pigmentosum [31, 43, 44], and dominant hereditary transmission of early adult pterygium [45]. The patient’s mother and sister had also pterygium. However, a genetic mutation analysis was not performed in our patient. Future studies with meticulous genetic mutation analysis and longer follow-up are recommended.

## CONCLUSION

We suggest vertical split simultaneous CAT as a safe and effective surgical technique for the treatment of recurrent double-headed pterygium. Family history, sunlight exposure, male gender and smoking were the main significant risk factors for pterygium in our patient. Postoperative medication is also important and using sunglasses for protection from ultraviolet should be advised to patients with pterygium to lessen the recurrence probability.

## DISCLOSURE

Ethical issues have been completely observed by the authors. All named authors meet the International Committee of Medical Journal Editors (ICMJE) criteria for authorship of this manuscript, take responsibility for the integrity of the work as a whole, and have given final approval for the version to be published. No conflict of interest has been presented. Funding/Support: None. The datasets analyzed during this study are available from the corresponding author on reasonable request.

## References

[1] Otulana TO (2012). Blindness and visual impairment in Remo, Ogun State, Nigeria: a hospital-based study. Niger Postgrad Med J.

[2] Xu L, Jonas JB, Cui TT, You QS, Wang YX, Yang H (2012). Beijing Eye Public Health Care Project. Ophthalmology.

[3] Elhamaky TR, Elbarky AM (2018). Outcomes of Vertical Split Conjunctival Autograft Using Fibrin Glue in Treatment of Primary Double-Headed Pterygia. J Ophthalmol.

[4] Duman F, Kosker M (2015). Demographics of Patients with Double-headed Pterygium and Surgical Outcomes. Turk J Ophthalmol.

[5] Yeung SN, Rubenstein D, Price AJ, Elbaz U, Zhang AQ, Cote E (2013). Sequential pterygium excision with conjunctival autograft in the management of primary double-headed pterygia. Can J Ophthalmol.

[6] Aidenloo NS, Motarjemizadeh Q, Heidarpanah M (2018). Risk factors for pterygium recurrence after limbal-conjunctival autografting: a retrospective, single-centre investigation. Jpn J Ophthalmol.

[7] Lee JS, Ha SW, Yu S, Lee GJ, Park YJ (2017). Efficacy and Safety of a Large Conjunctival Autograft for Recurrent Pterygium. Korean J Ophthalmol.

[8] Janson BJ, Sikder S (2014). Surgical management of pterygium. Ocul Surf.

[9] Wolter-Roessler E, Seitz B, Naumann GO (2002). [Pterygoid corneal dystrophy]. Klin Monbl Augenheilkd.

[10] Chen T, Ding L, Shan G, Ke L, Ma J, Zhong Y (2015). Prevalence and racial differences in pterygium: a cross-sectional study in Han and Uygur adults in Xinjiang, China. Invest Ophthalmol Vis Sci.

[11] Li Z, Wu S, Mai J, Xu K, Sun Y, Song Z (2014). Prevalence of and risk factors for pterygia in a rural Northern Chinese population. Ophthalmic Epidemiol.

[12] Jiao W, Zhou C, Wang T, Yang S, Bi H, Liu L (2014). Prevalence and risk factors for pterygium in rural older adults in Shandong Province of China: a cross-sectional study. Biomed Res Int.

[13] Nangia V, Jonas JB, Nair D, Saini N, Nangia P, Panda-Jonas S (2013). Prevalence and associated factors for pterygium in rural agrarian central India The central India eye and medical study. PLoS One.

[14] Liu L, Wu J, Geng J, Yuan Z, Huang D (2013). Geographical prevalence and risk factors for pterygium: a systematic review and meta-analysis. BMJ Open.

[15] Altinkaynak H, Demircan A, Kocasarac C, Kara N, Dundar H, Altan C (2014). Effect of orbital protrusion and vertical interpalpebral distance on pterygium formation. Cont Lens Anterior Eye.

[16] Rezvan F, Hashemi H, Emamian MH, Kheirkhah A, Shariati M, Khabazkhoob M (2012). The prevalence and determinants of pterygium and pinguecula in an urban population in Shahroud, Iran. Acta Med Iran.

[17] Li Z, Cui H (2013). Prevalence and associated factors for pterygium in a rural adult population (the Southern Harbin Eye Study). Cornea.

[18] Cajucom-Uy H, Tong L, Wong TY, Tay WT, Saw SM (2010). The prevalence of and risk factors for pterygium in an urban Malay population: the Singapore Malay Eye Study (SiMES). Br J Ophthalmol.

[19] Durkin SR, Abhary S, Newland HS, Selva D, Aung T, Casson RJ (2008). The prevalence, severity and risk factors for pterygium in central Myanmar: the Meiktila Eye Study. Br J Ophthalmol.

[20] Lu J, Wang Z, Lu P, Chen X, Zhang W, Shi K (2009). Pterygium in an aged Mongolian population: a population-based study in China. Eye (Lond).

[21] Tan CS, Lim TH, Koh WP, Liew GC, Hoh ST, Tan CC (2006). Epidemiology of pterygium on a tropical island in the Riau Archipelago. Eye (Lond).

[22] Gazzard G, Saw SM, Farook M, Koh D, Widjaja D, Chia SE (2002). Pterygium in Indonesia: prevalence, severity and risk factors. Br J Ophthalmol.

[23] Wong TY, Foster PJ, Johnson GJ, Seah SK, Tan DT (2001). The prevalence and risk factors for pterygium in an adult Chinese population in Singapore: the Tanjong Pagar survey. Am J Ophthalmol.

[24] Maharshak I, Avisar R (2009). Bilateral primary pterygia: an occupational disease?. Arch Environ Occup Health.

[25] Akinsola FB, Mbadugha CA, Onakoya AO, Adefule-Ositelu AO, Aribaba OT, Rotimi-Samuel A (2012). Pattern of conjunctival masses seen at Guinness Eye Centre Luth Idi-Araba. Nig Q J Hosp Med.

[26] Malekifar P, Esfandiari H, Behnaz N, Javadi F, Azish S, Javadi MA (2017). Risk Factors for Pterygium in Ilam Province, Iran. J Ophthalmic Vis Res.

[27] Roka N, Shrestha SP, Joshi ND (2013). Assessment of tear secretion and tear film instability in cases with pterygium and normal subjects. Nepal J Ophthalmol.

[28] Salustiano Correa ESR, de Pereira Avila M, Rassi AR, Ximenes L, da Silva DS Jr, de Paula AC (2013). Intra-operative use of 5-Fluorouracil in pterygium surgery: a comparative study. Semin Ophthalmol.

[29] Kareem AA, Farhood QK, Alhammami HA (2012). The use of antimetabolites as adjunctive therapy in the surgical treatment of pterygium. Clin Ophthalmol.

[30] Huerva V, March A, Martinez-Alonso M, Muniesa MJ, Sanchez C (2012). Pterygium surgery by means of conjunctival autograft: long term follow-up. Arq Bras Oftalmol.

[31] Kobayashi A, Shirao Y, Segawa Y, Higashide T, Miwa S, Kawasaki K (2001). Multi-layer amniotic membrane graft for pterygium in a patient with xeroderma pigmentosum. Jpn J Ophthalmol.

[32] Gonzalez Gomez A, Gonzalez de Gor Crooke JL, Garcia-Ben A, Garcia-Campos JM (2015). Dellen and corneal perforation after bilateral pterygium excision in a patient with no risk factors. BMJ Case Rep.

[33] Salagar KM, Biradar KG (2013). Conjunctival autograft in primary and recurrent pterygium: a study. J Clin Diagn Res.

[34] Fernandes M, Sangwan VS, Bansal AK, Gangopadhyay N, Sridhar MS, Garg P (2005). Outcome of pterygium surgery: analysis over 14 years. Eye (Lond).

[35] Das S, Ramamurthy B, Sangwan VS (2009). Deep lamellar keratoplasty for recurrent advanced pterygium. Ophthalmic Surg Lasers Imaging.

[36] Sudhalkar A (2012). Fixation and its role in the causation, laterality and location of pterygium: a study in amblyopes and non-amblyopes. Eye (Lond).

[37] Ciftci S (2009). Bilateral pterygium, symmetrical nodosity of the auricle, and free iris cyst. Can J Ophthalmol.

[38] Demirok A, Cinal A, Yener HI, Yasar T, Kilic A (2008). The risk factors of pterygium development: a hospital-based study. Ann Ophthalmol (Skokie).

[39] Yalcin Tok O, Burcu Nurozler A, Ergun G, Akbas Kocaoglu F, Duman S (2008). Topical cyclosporine A in the prevention of pterygium recurrence. Ophthalmologica.

[40] Ukponmwan CU, Dawodu OA, Edema OF, Okojie O (2007). Prevalence of pterygium and pingueculum among motorcyclists in Nigeria. East Afr Med J.

[41] Taylor SL, Coates ML, Vallejos Q, Feldman SR, Schulz MR, Quandt SA (2006). Pterygium among Latino migrant farmworkers in North Carolina. Arch Environ Occup Health.

[42] Molho-Pessach V, Mechoulam H, Siam R, Babay S, Ramot Y, Zlotogorski A (2015). Ophthalmologic Findings in H Syndrome: A Unique Diagnostic Clue. Ophthalmic Genet.

[43] Emmert S, Ueda T, Zumsteg U, Weber P, Khan SG, Oh KS (2009). Strict sun protection results in minimal skin changes in a patient with xeroderma pigmentosum and a novel c2009delG mutation in XPD (ERCC2). Exp Dermatol.

[44] Goyal JL, Rao VA, Srinivasan R, Agrawal K (1994). Oculocutaneous manifestations in xeroderma pigmentosa. Br J Ophthalmol.

[45] Hecht F, Shoptaugh MG (1990). Winglets of the eye: dominant transmission of early adult pterygium of the conjunctiva. J Med Genet.

